# Optimizing the Eden-Hybinette Procedure With 3D-Printed Patient-Specific Instrumentation: A Technical Note

**DOI:** 10.7759/cureus.95817

**Published:** 2025-10-31

**Authors:** Bernard de Geofroy, Damien Lami, Anne-Sophie Lamort, Jean-Charles Grillo, Camille Choufani

**Affiliations:** 1 Orthopaedics and Traumatology, Laveran Military Hospital, Marseille, ATF; 2 Orthopaedic Surgery, Institut du Mouvement et de l'Appareil Locomoteur, Marseille, FRA; 3 Laboratory, Newclip Technics, Haute Goulaine, FRA; 4 Orthopaedics and Traumatology, Pole ORTHO-SPORT Les Fleurs, Toulon, FRA; 5 Orthopaedics and Traumatology, Sainte-Anne Military Hospital, Toulon, FRA

**Keywords:** 3d printing technology, anterior shoulder instability, eden-hybinette procedure, iliac crest bone graft, patient-specific instrumentation (psi)

## Abstract

The Eden-Hybinette procedure using an iliac crest bone graft is indicated in cases of recurrent anterior shoulder instability with major glenoid bone loss (>40%), coracoid dysplasia, or failure of Latarjet or Bristow-Latarjet procedures. We describe an open Eden-Hybinette technique supported by a 3D-printed patient-specific cutting guide for iliac crest graft harvesting, combined with a dedicated positioning device (HyLa, Hybrid Latarjet, Newclip Technics®). Preoperative planning based on CT scans allows precise assessment of glenoid bone loss, calculation of graft dimensions, and determination of optimal screw length and orientation. The patient-specific guide enables reproducible tricortical graft harvesting, while the HyLa device provides intraoperative control of graft positioning, avoiding graft overhang or medialization. The technique can be performed either open or arthroscopically. The use of patient-specific instrumentation allowed accurate graft sizing and placement, with postoperative imaging confirming graft congruency and fixation stability. Patients resumed physical activity within six months without recurrence of instability or donor-site morbidity. This technical note highlights the potential advantages of combining 3D-printed patient-specific guides with dedicated instrumentation for the Eden-Hybinette procedure. Anticipating graft size, shape, and screw trajectory may improve accuracy, reproducibility, and safety in revision shoulder stabilization. Further clinical studies are required to validate outcomes compared with conventional techniques.

## Introduction

Anterior shoulder instability is a frequent condition in young adults, particularly in contact athletes and military populations [1]. It results from repetitive trauma or a single dislocation event leading to glenoid bone loss and capsulolabral insufficiency, both of which compromise joint stability. The Latarjet procedure is a well-established surgical solution [2], providing excellent clinical outcomes [3] and high return-to-sport rates [4]. However, recurrence of instability after coracoid transfer has been reported in 7% to 11% of cases [5], with most failures occurring within the first postoperative year (73%) [6]. In such cases, revision with the Eden-Hybinette procedure using an iliac crest bone graft is the most commonly employed technique, yielding satisfactory clinical results [7]. Three-dimensional (3D) printing and patient-specific instrumentation (PSI) are increasingly applied in orthopedic surgery [8]. Several studies have demonstrated improved implant positioning in hip and knee procedures with PSI [9], and this technology is now being adopted in shoulder surgery as well [10]. In the Eden-Hybinette procedure, both graft harvesting and graft positioning may potentially be optimized using patient-specific cutting guides. To date, however, no study has described the use of PSI in this context.

This technical report aims to describe an open Eden-Hybinette procedure using a 3D-printed patient-specific iliac crest cutting guide, combined with a dedicated positioning device (HyLa, Hybrid Latarjet, Newclip Technics®, Haute-Goulaine, France), specifically designed to optimize graft harvesting and placement in cases of failed Latarjet procedures.

## Technical report

Concept and materials

**Figure 1:**
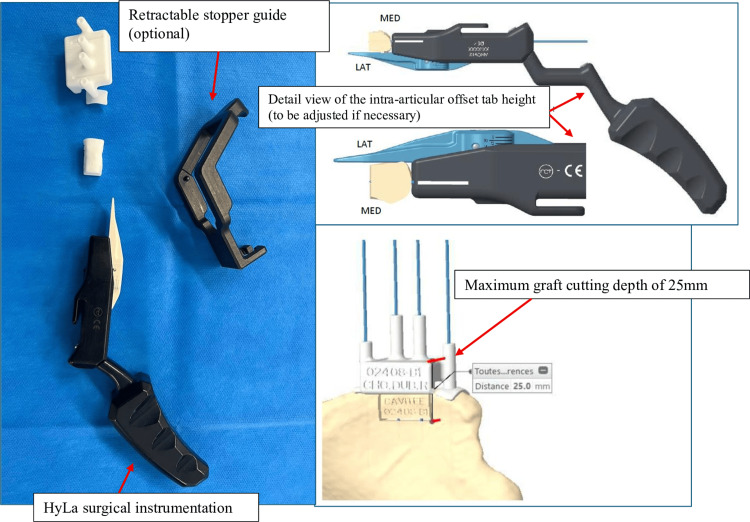
Patient-specific instrumentation for the Eden-Hybinette technique. Custom-made 3D-printed guide used for accurate graft positioning in the Eden-Hybinette procedure.

Using the commercially available HyLa instrumentation described by Lami et al. [11], which allows an anterior shoulder bone block according to the Latarjet procedure by combining open harvesting with arthroscopic graft fixation, we adapted this concept for the Eden-Hybinette iliac crest bone block. The innovation consists of a patient-specific cutting guide enabling standardized graft harvesting, tailored to both glenoid morphology and the HyLa positioning device (Figure 1).

This approach was designed to address several potential pitfalls, including inadequate graft size, which increases the risk of fracture during screw fixation if the graft is too small; poor graft morphology, leading to suboptimal adaptation to the glenoid contour; malposition of the bone block, resulting in either excessive lateral overhang or undue medialization; and the use of excessively long screws, which may cause posterior conflict. The expected benefits of this technique include standardized graft harvesting, with dimensions and morphology adapted to the glenoid, assessment of bone loss, and adjustment based on the Glenoid Track measurement [12], thereby minimizing the risk of malpositioning. Pre-positioning of guide pins during graft harvesting allows reproducible screw spacing and placement within the graft. Graft positioning is further optimized by the adjustable intra-articular blade of the HyLa device, which prevents overhang while maintaining fixation through a two-in-one system. Finally, anticipation of screw length helps avoid anterior-posterior protrusion and reduces the risk of subscapularis muscle conflict. This article describes the open version of the procedure.

Preoperative planning and guide design

**Figure 2:**
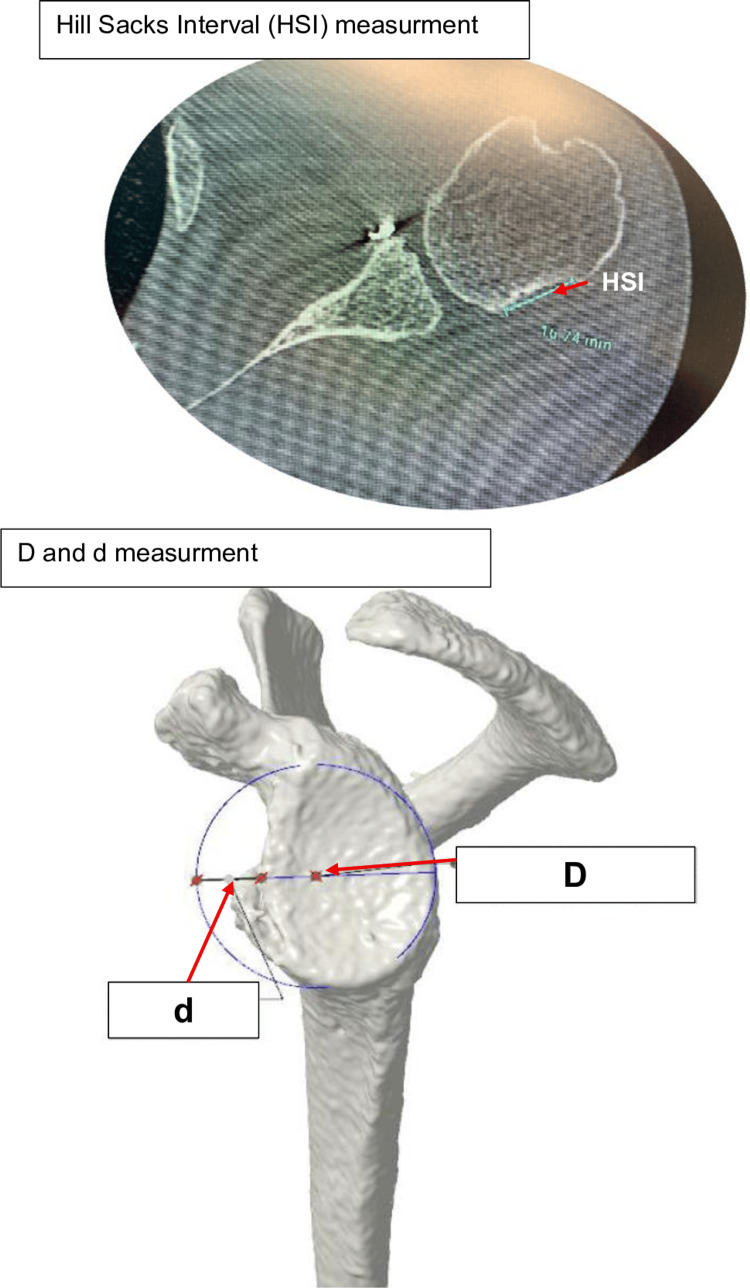
Measurement of glenohumeral tracking. Evaluation of glenohumeral joint kinematics to ensure correct alignment and stability.

DICOM (Digital Imaging and Communications in Medicine) files from preoperative CT scans of the shoulder and pelvis were obtained to create 3D reconstructions of the bony surfaces of the glenoid and iliac crest. These data were processed using ONE software (Newclip®, Haute-Goulaine, France). Virtual placement of an iliac crest bone block was performed on the anterior glenoid to determine optimal graft size and shape. Several key measurements were obtained, including the Glenoid Track (GT = 0.83 × D - d), Hill-Sachs Interval (HSI), diameter of the “best fit circle” of the glenoid (D), and the width of the anterior glenoid defect (d) (Figure 2).

Formulas Applied

Formula 1: D2 = D - d + E - 2.

Formula 2: GT2 = 0.83 × D2 - d.

Formula 3: GT = 0.83 × D - d.

(D2 = “best fit circle” of the glenoid after bone block; GT2 = Glenoid Track after bone block; E = graft thickness).

Objective: GT2 > HSI (to be considered “on-track”); therefore, E > (HSI - d)/0.83 + 2 - D + d.

Based on these calculations, the cutting guide was modeled to match the bone defect. Its positioning on the iliac crest was adapted to allow harvesting of a tricortical graft of appropriate size. Landmarks such as the anterior superior iliac spine (ASIS) were used to ensure reproducibility intraoperatively.

Simulation of graft placement also allowed preoperative determination of optimal screw length to ensure bicortical fixation on the glenoid. Once validated by the surgeon, the customized iliac crest cutting guide was 3D-printed and sterilized for surgical use.

Operative technique

**Figure 3:**
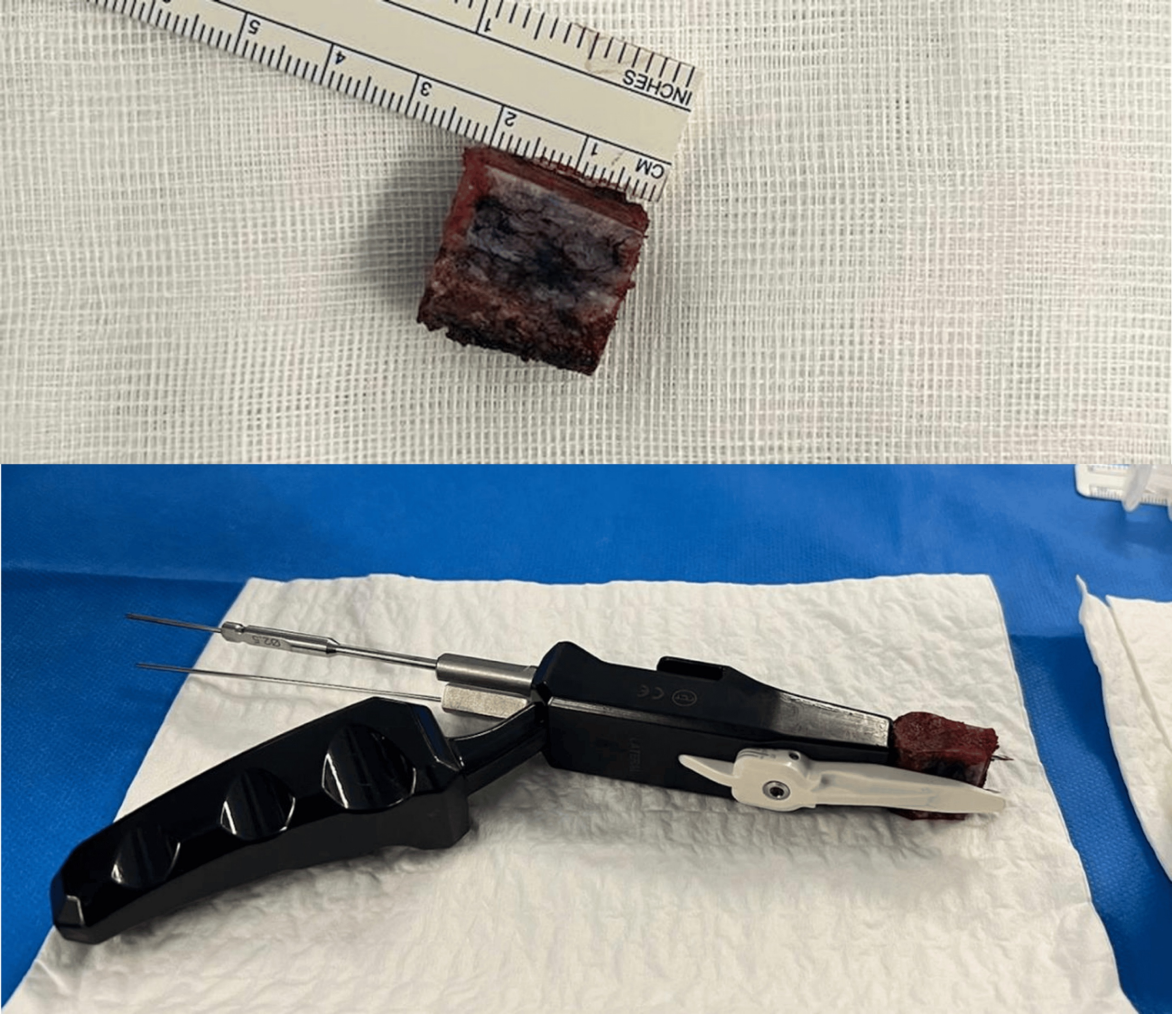
Graft adaptation on the HyLa instrumentation set. Adjustment of the iliac crest graft using the HyLa surgical instrumentation.

The procedure is performed under general anesthesia with an interscalene block. The patient is placed supine, with both the operative shoulder and ipsilateral iliac crest prepped and draped. An incision is made 2 cm posterior to the ASIS, along the iliac crest. The abdominal oblique and transverse muscles are detached. The patient-specific guide is positioned on the superior iliac crest according to the preoperative plan, with a reference distance from the ASIS. The guide is fixed with four 1.2 mm pins, enabling the three cuts required for tricortical graft harvesting. The two superior pins are left in place to allow secure fixation of the graft within the HyLa positioning device. Before removing the graft, drilling is performed along the guide pins to prepare the screw trajectories (Figure 3).

**Figure 4:**
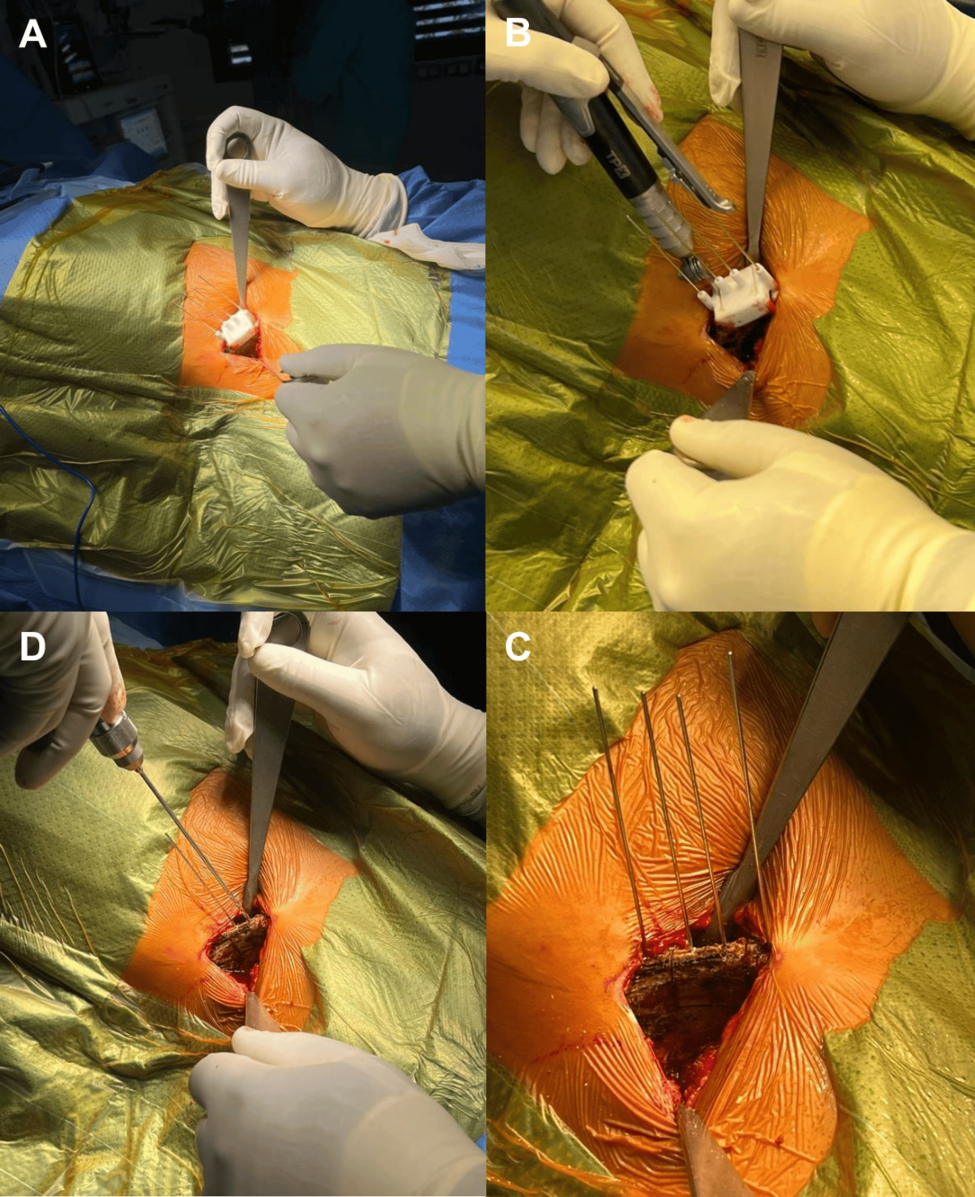
Four steps of iliac crest graft harvesting with a 3D-printed cutting guide. (A) Placement of the custom guide fixed with four pins. (B) Oscillating saw cut to shape the graft. (C) Drilling of the two screw holes guided by the central pins. (D) Final graft resection, with the central pins maintained to secure the graft on the HyLa instrumentation set.

A deltopectoral approach is performed, reusing the previous incision in revision cases. Careful dissection is performed to avoid brachial plexus injury, with exposure of the superior and inferior borders of the subscapularis. A split of the subscapularis is made along the fiber axis at its midportion. Intra-articular visualization of the anteroinferior glenoid is achieved with a Fukuda retractor. Removal of previous fixation (screws or buttons) may be required. The iliac crest bone block, previously mounted on the HyLa device with the predefined orientation, is positioned onto the anteroinferior glenoid, just below the equator (Figure 4).

**Figure 5:**
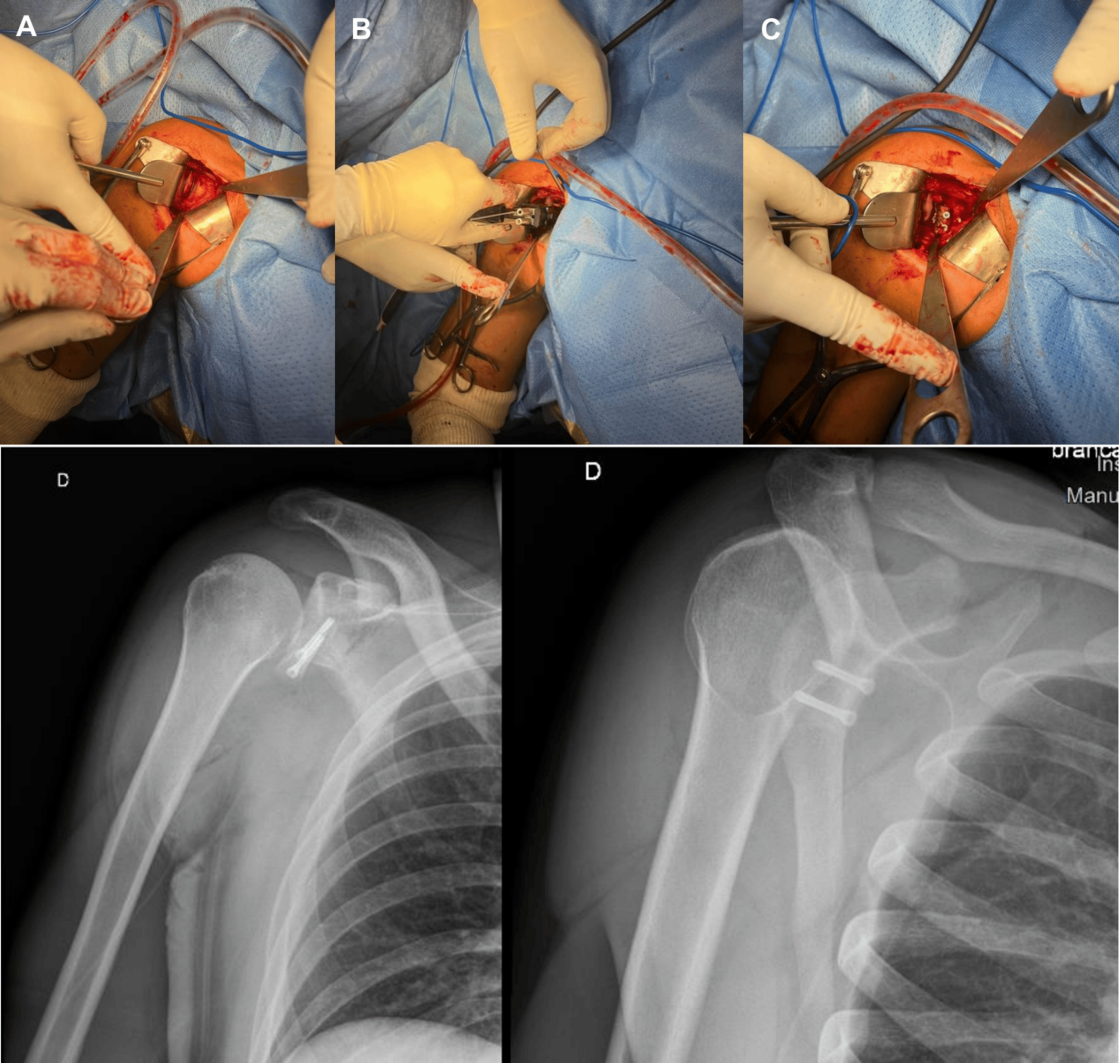
Graft placement with the HyLa instrumentation and postoperative radiographs. Final positioning and fixation of the iliac crest graft using the HyLa surgical set, with postoperative radiographs confirming proper graft alignment and fixation. (A) Exposure of the anteroinferior glenoid rim. (B. Positioning of the bone-block instrumentation at the anteroinferior glenoid rim. (C, D) Fixation of the graft with two screws.

The adjustable intra-articular blade of the HyLa device prevents graft overhang. The two guide pins are advanced into the glenoid, and unicortical drilling is performed to facilitate insertion of two self-tapping, self-drilling countersunk screws (3.5 mm) of predetermined length based on the 3D plan (Figure 5).

Table 1 presents a summary of the five military patients who underwent revision surgery for recurrent anterior shoulder instability using the Eden-Hybinette procedure with 3D-printed PSI. The table details demographic data, graft dimensions, screw length, and postoperative outcomes. All patients returned to work and physical activity within six months, with no recurrence of instability or donor-site morbidity observed.

**Table 1:** Patient demographics, surgical parameters, and postoperative outcomes following the Eden-Hybinette procedure using 3D-printed patient-specific instrumentation.

Patient	Age (years)	Dominant side	Sport/Occupation	Indication for revision	Graft dimensions (mm)	Screw length (mm)	Return to work	Return to physical activity	Complications
1	28	Right	Military/Contact sports	Recurrent instability after Latarjet	25 × 10 × 8	36	6 months	7 months	None
2	32	Right	Military/Gym training	Coracoid graft fracture	26 × 11 × 9	38	5 months	6 months	Postoperative hematoma
3	30	Left	Military/Rugby	Glenoid bone loss >40%	27 × 12 × 8	40	6 months	5 months	None
4	27	Right	Military/Obstacle course	Failed fixation (screw loosening)	24 × 10 × 7	34	5 months	6 months	None
5	29	Right	Military/Combat training	Persistent pain and instability	25 × 11 × 8	38	6 months	7 months	None

## Discussion

Postoperative radiographs were obtained to confirm appropriate graft positioning. The rehabilitation protocol allowed immediate shoulder mobilization with pendulum exercises, followed by passive range of motion at three weeks and active motion at six weeks. All five military patients successfully returned to work at six months, which corresponds to the expected graft consolidation period, and resumed physical activity without discomfort, even during demanding exercises such as push-ups and pull-ups. Notably, no pain or morbidity was reported at the iliac crest donor site, underlining the minimally invasive nature of this procedure. Postoperative follow-up was favorable, with no recurrence of instability or shoulder subluxation. Glenohumeral congruency was preserved, as confirmed by postoperative CT scan, supporting the overall success of this procedure from surgical execution to functional recovery. The Eden-Hybinette iliac crest bone block technique, initially described more than 100 years ago by Eden and Hybinette [13], has long been used for revision of failed Latarjet procedures [14]. Several modifications have been reported [15], culminating in the development of fully arthroscopic techniques [16]. The indications for Eden-Hybinette have since expanded, including use as an alternative to Latarjet [17] and in cases of instability associated with epilepsy [18]. With the recent evolution of computer-assisted and patient-specific surgery, multiple techniques have been developed to simplify and secure surgical procedures [19]. The Latarjet procedure was the first in shoulder stabilization to benefit from this innovation. Doursounian et al. (2009) reported that PSI improved reproducibility and simplified graft placement in Latarjet surgery [20]. More recently, La Banca et al. described the use of 3D-printed patient-specific guides for coracoid harvesting and positioning during the Latarjet procedure [10]. Compared with the existing literature, the innovation of our technique lies in extending the application of PSI to the Eden-Hybinette procedure. The anticipated benefits mirror those observed in other orthopedic procedures: greater accuracy, reproducibility, and safety. Furthermore, patient-specific planning enables surgeons to anticipate potential difficulties, such as unexpected glenoid bone loss or atypical iliac crest morphology, which may otherwise complicate surgery. Finally, the integration of glenohumeral tracking analysis provides additional support for graft dimensioning. By using a custom 3D cutting guide, the iliac crest graft can be harvested with the exact dimensions planned preoperatively, ensuring optimal fit and positioning during fixation.

## Conclusions

The combination of 3D preoperative planning and PSI described in this technical note offers a reproducible and precise method for performing the Eden-Hybinette procedure. The integration of customized cutting and positioning guides enhances the accuracy of graft harvesting and placement, while maintaining surgical safety and efficiency. These preliminary results support the feasibility and potential of this approach as a valuable adjunct in complex revision cases of anterior shoulder instability. However, given the limited number of patients and short-term follow-up, these findings should be interpreted with caution. Further prospective and comparative studies are needed to validate long-term outcomes, particularly regarding graft integration, glenohumeral stability, and cost-effectiveness compared with conventional techniques.
